# Characterization of *bla*_OXA-48_-carrying plasmids and small non-AMR-coding plasmids collected from Ukrainian patients

**DOI:** 10.1007/s15010-023-02136-2

**Published:** 2023-11-29

**Authors:** Vincent F. van Almsick, Annika Sobkowiak, Natalie Scherff, Franziska Schuler, Alexander Mellmann, Vera Schwierzeck

**Affiliations:** 1 Institute of Hygiene, University Hospital Münster, Robert-Koch-Straße 41, Münster, Germany; 2 Institute of Medical Microbiology, University Hospital Münster, Münster, Germany; 3 Department of Cardiology I – Coronary and Peripheral Vascular Disease, Heart Failure, University Hospital Münster, Münster, Germany

**Keywords:** Long-read whole genome sequencing, Multi-drug resistance bacteria, Antimicrobial resistance, *bla*_OXA-48_, Plasmids

## Abstract

**Purpose:**

Carbapenemase-producing Enterobacterales (CPE) pose a serious threat for healthcare facilities worldwide, yet the mode of transmission is often unclear. Recently, we recorded an increase of *bla*_OXA-48_-harboring isolates at our hospital associated with patients with previous medical treatment in the Ukraine. We used long-read whole genome sequencing (lrWGS) to characterize these isolates including their plasmids.

**Methods:**

Samples were collected as part of clinical routine diagnostic or screening of multi-drug resistance bacteria (MDRB). Antimicrobial susceptibility testing was performed and all MDRB (*n* = 10) were sequenced by lrWGS for genotyping, identification of antimicrobial resistance (AMR) genes, and characterization of plasmids.

**Results:**

While routine analysis of core genome multilocus sequence typing (cgMLST) did not show any genetic similarities between isolates, we found an unexpected high similarity in the plasmid diversity of different Enterobacterales in patients with previous medical treatment in the Ukraine. This included an IncL/M plasmid carrying *bla*_OXA-48_ and additional small non-AMR-coding plasmids.

**Conclusion:**

Our results show that lrWGS can be used in the routine setting to uncover similarities in plasmids and may give further information about potential epidemiologic associations. In the future, analysis of both AMR and non-AMR plasmids may provide an additional layer of information for molecular surveillance of CPE.

**Supplementary Information:**

The online version contains supplementary material available at 10.1007/s15010-023-02136-2.

## Introduction and purpose

Carbapenemase-producing Enterobacterales (CPE) are still rare in our 1,500-bed university hospital in the northwestern part of Germany. However, in 2022, we noticed an increase of *bla*_OXA-48_-harboring isolates in screening and clinical samples compared to previous years. To rule out intrahospital transmission of CPE, we reviewed the case history of the affected patients (PatA and PatB, Table 1) and realized that all *bla*_OXA-48_-harboring isolates in 2022 were associated with Ukrainian refugees admitted to our hospital for medical emergency treatment. In fact, other publications have described similar findings, suggesting a high prevalence of *bla*_OXA-48_-carrying Enterobacterales in the Ukrainian healthcare system at present [1, 2]. We performed long-read whole genome sequencing (lrWGS) of all *bla*_OXA-48_-harboring isolates and of all ESBL-producing Enterobacterales (*n* = 5) from these patients. As part of routine surveillance, we sequence all MDRB isolates using lrWGS at the time of first detection at our hospital; therefore, we were able to compare the isolates associated with the Ukraine and their plasmids with other samples with IncL/M plasmids from our hospital (*n* = 5). Moreover, we included previously well-characterized plasmids from the literature as comparison.

**Table 1:**
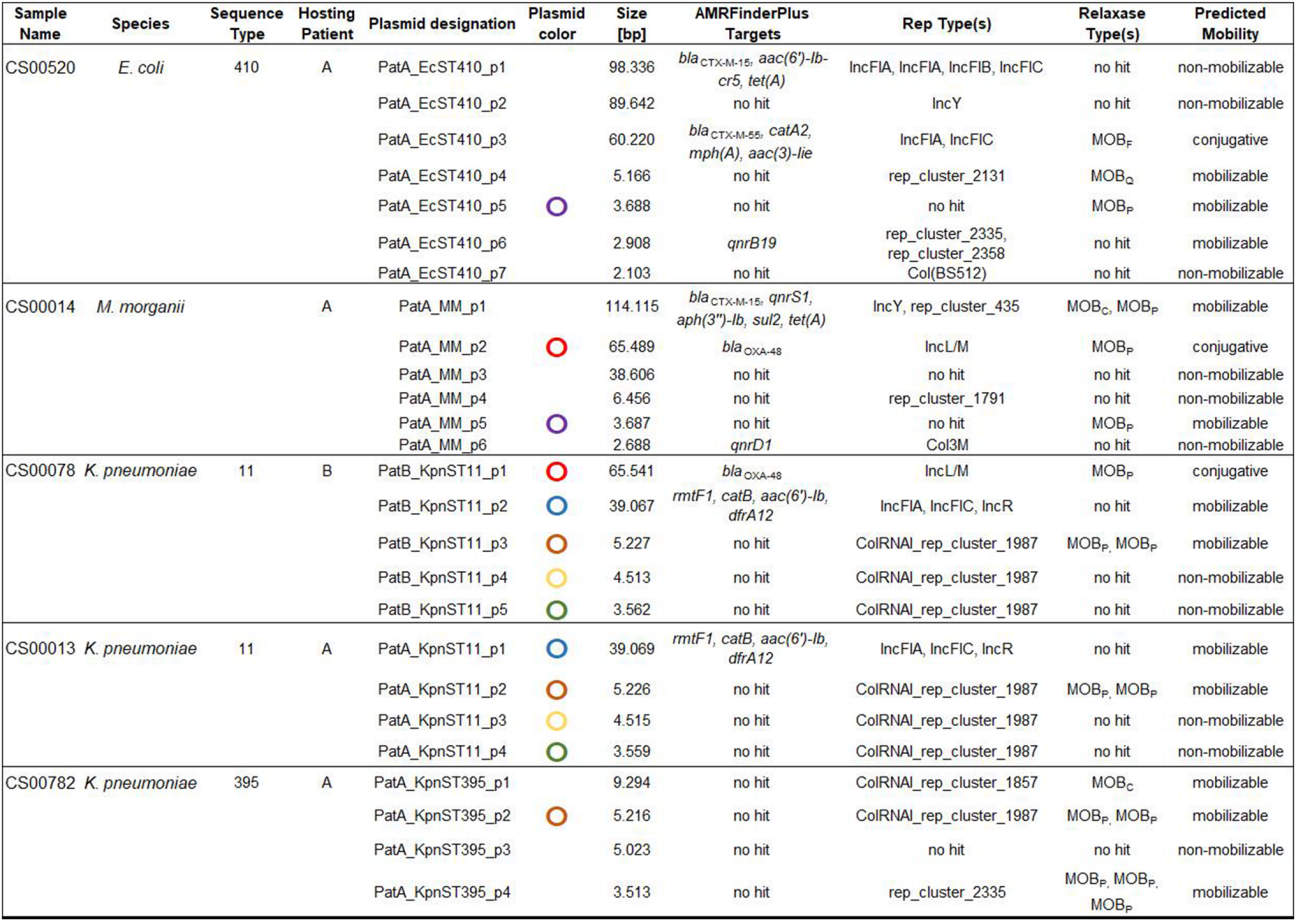
Molecular characterization of bacterial isolates with suggested horizontal plasmid transfer including their circular plasmids and their classification using the MOB suite

Different plasmid clusters with 100% overlap in Blast analysis are indicated by same plasmid color (Blast comparison is shown in Supplementary_Figure B)

## Methods

In addition to the detection of multi-drug resistance bacteria (MDRB) from clinical samples, anorectal swab samples were collected as part of routine surveillance for multi-drug-resistant Gram negative bacteria. Screening samples were cultivated on chromID® ESBL Agar (biomérieux, Marcy-l’Etoile, France), CHROMagar™ Acinetobacter (Mast Group, Paris, France), and Pseudomonas Cetrimide Agar (Thermo Fisher Scientific, Basingstoke, UK). Species identification was performed by matrix-assisted laser desorption/ionization time-of-flight mass spectrometry (MALDI-TOF/MS) (Bruker, Bremen, Germany). Antimicrobial susceptibility testing was performed using VITEK® 2 (biomérieux, Marcy-l’Etoile, France) and Etest® (biomérieux, Marcy-l’Etoile, France). Antibiotic susceptibility was interpreted according to the 2022 European Committee on Antimicrobial Susceptibility Testing (EUCAST, v.12.0) criteria.

Genomic DNA (gDNA) from bacterial isolates was extracted using the NEB Monarch Genomic Purification Kit (New England Biolabs, Ipswich, MA, USA). All isolates were sequenced on a PacBio® Sequel IIe system (Pacific Biosciences, Menlo Park, CA, USA). Next, raw sequences were assembled de novo and analyzed using the SMRT® Link software suite v.10 or v.11 with default parameters. Isolates were genotyped based on core genome multilocus sequence typing (cgMLST) targets implemented in Ridom SeqSphere^+^ software version 8.99 (Ridom GmbH, Münster, Germany) [3]. In addition, the MLST sequence types (ST) were identified using the schemes for the respective species. Antimicrobial resistance (AMR) genes and their location were determined using target gene sets for antimicrobial resistance based on the NCBI AMRFinderPlus [4]. To predict the plasmid replicon type, the respective contigs were checked for complete circularization and analyzed with MobSuite [5]. For annotation, we used the online pipeline dfast (including HMM scan against TIGRFAM and RPSBLAST against COG database from NCBI) [6]. We performed BLAST analyses using BRIG Software (BLAST Ring Image Generator) [7]. Same secondary cluster ID by MobSuite was the indicator for us to perform BLASTn analysis via BRIG Software.

## Results

**Fig. 1:**
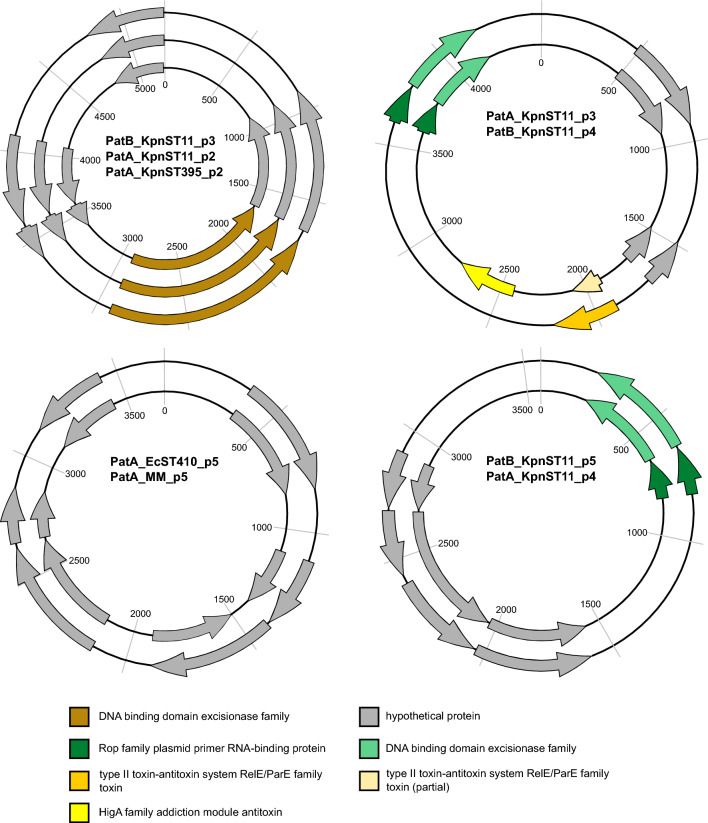
Genetic content of the four SCP clusters. Kpn = *K. pneumoniae*, MM = *M. morganii*, Ec = *E. coli*, ST = MLST sequence type. The full names of the compared plasmids are written in the center of the plasmid rings. Names and further information related to the plasmids and patients are listed in Table 1. Hypothetical proteins are colored in grey; identical colors indicate same coding proteins

At first glance, cgMLST did not reveal any significant genetic relation among the five Ukrainian isolates. The closest genetic relationship observed was between two ST11 *Klebsiella pneumoniae* isolates with an allelic distance of 11 and no documented contact during hospitalization. Next, we investigated the plasmid content of the isolates in the same patient based on lrWGS data (shown in Table 1). Interestingly, we identified an identical *bla*_OXA-48_ plasmid in two isolates, a *K.* *pneumoniae* and a *Morganella morganii.* The plasmid is classified by MobSuite as an IncL/M plasmid with a length of 65,540 bp. Within this plasmid, a resistance cassette with a *bla*_OXA-48_ AMR gene was detected. As the plasmid is classified as conjugative and shows 100% overlap in the Blast analysis (Supplementary_Figure B, plasmids PatA_MM_p2 and PatB_KpnST11_p1) in isolates of two different species, we assume horizontal gene transfer has occurred at some point in the past. This is consistent with the already known high rate of conjugation in IncL/M-*bla*_OXA-48_ positive plasmids [8]. A deeper view into the case history of the patients showed that they had escaped the Ukraine because of the Russian invasion and both had previous treatment in the region of Charkiw in 2022. To estimate how prevalent this plasmid is in the bacterial population, we compared the respective plasmids with another five IncL/M plasmids, all of which were collected in our hospital from 2022 to April 2023 (see Supplementary_Figure A). No IncL/M plasmid with the same degree of similarity was identified. However, in a Blast comparison with the NCBI database, we found an identical IncL/M plasmid with the same AMR gene collected at the Charles University in Prague, Plzen, Czech Republic in 2016 (SAMN14227071) (see Supplementary_Figure A). As a next step, we compared all the non-*bla*_OXA-48_-containing plasmids of the five isolates associated with the Ukraine, using MobSuite based on a mash-distance analysis [9]. Interestingly, characterization of small cryptic plasmids (SCPs) (< 10 kb) [10] revealed additional links among the isolates. Here we detected four clusters with nine plasmids in the Ukrainian isolates. We found no SCP cluster in the isolates from our hospital. This observation also suggests ongoing plasmid exchange among bacteria in the same health region, as the co-conjugation of SCPs has already been shown in the case of AMR plasmid transmission [10]. So far the function of these small plasmids (Fig. 1) (3.5 kb, 3.6 kb, 4.5 kb and 5.2 kb) is unclear, as they only encode a few proteins. The 3.5 kb plasmid contains three hypothetical proteins and a Rop family plasmid primer RNA-binding protein; the 4.5 kb plasmid encodes for three hypothetical proteins, a Rop family plasmid primer RNA-binding protein, and a type II toxin-antitoxin system RelE/ParE family toxin. The 5.2 kb plasmid harbors four hypothetical proteins and a DNA cytosine methyltransferase and the 3.6 kb plasmid only for hypothetical proteins (Annotation and comparison is shown in Fig. 1, BLAST analysis in Supplementary_Figure B).

## Conclusion

As the characterization of AMR plasmids is not part of routine surveillance efforts in hospitals, it is still unclear how AMR-specific plasmids are disseminated in the health care setting. Our results suggest that the plasmid diversity of bacterial isolates may be part of the local epidemiology of MDRB. As lrWGS becomes more common in molecular diagnostics and surveillance, analyzing AMR plasmids in more detail in the hospital setting will uncover horizontal transmission events and might occasionally give us clues to the travel history of their hosts or connect isolates with each other. So far, little is known about the function of small non-AMR coding plasmids, yet small non-AMR-coding plasmids may give us more information about epidemiologic links.

### Supplementary Information

Below is the link to the electronic supplementary material.

Supplementary file1 (JPG 257 KB) Supplementary_Figure: Comparisons using BLAST analysis and BRIG software for imaging of IncL/M plamids. A) The IncL/M blaOXA-48 positive plasmids associated with patients from the Ukraine are colored in red, all other IncL/M plasmids found in our hospital in 2022 until April 2023 are colored in blue. As comparison, a reference plasmid from the NCBI database (GenBank accession number KX523901.1) was added (yellow)

Supplementary file2 (JPG 243 KB) Supplementary_Figure: Comparisons using BLAST analysis and BRIG software for imaging of IncL/M plamids. B) Blast comparisons of all detected plasmids. Color-coding was done according to Table 1: The IncL/M blaOXA-48 positive plasmids are colored red. An IncF/IncR plasmid is colored blue (PatA_KpnST11_p1 and PatB_KpnST11_p2). The four remaining plasmid groups colored in yellow, purple, orange and green belong to the SCP clusters (also see Figure 1)

## Data Availability

All samples are submitted to NCBI in BioProject PRJNA972998.
