# Supplementary figures and images for: Characterization of blaOXA-48-carrying plasmids and small non-AMR-coding plasmids collected from Ukrainian patients
Source: Infection. 2023 Nov 29;52(2):661–5. doi: 10.1007/s15010-023-02136-2 (PMC10954994; doi:10.1007/s15010-023-02136-2)

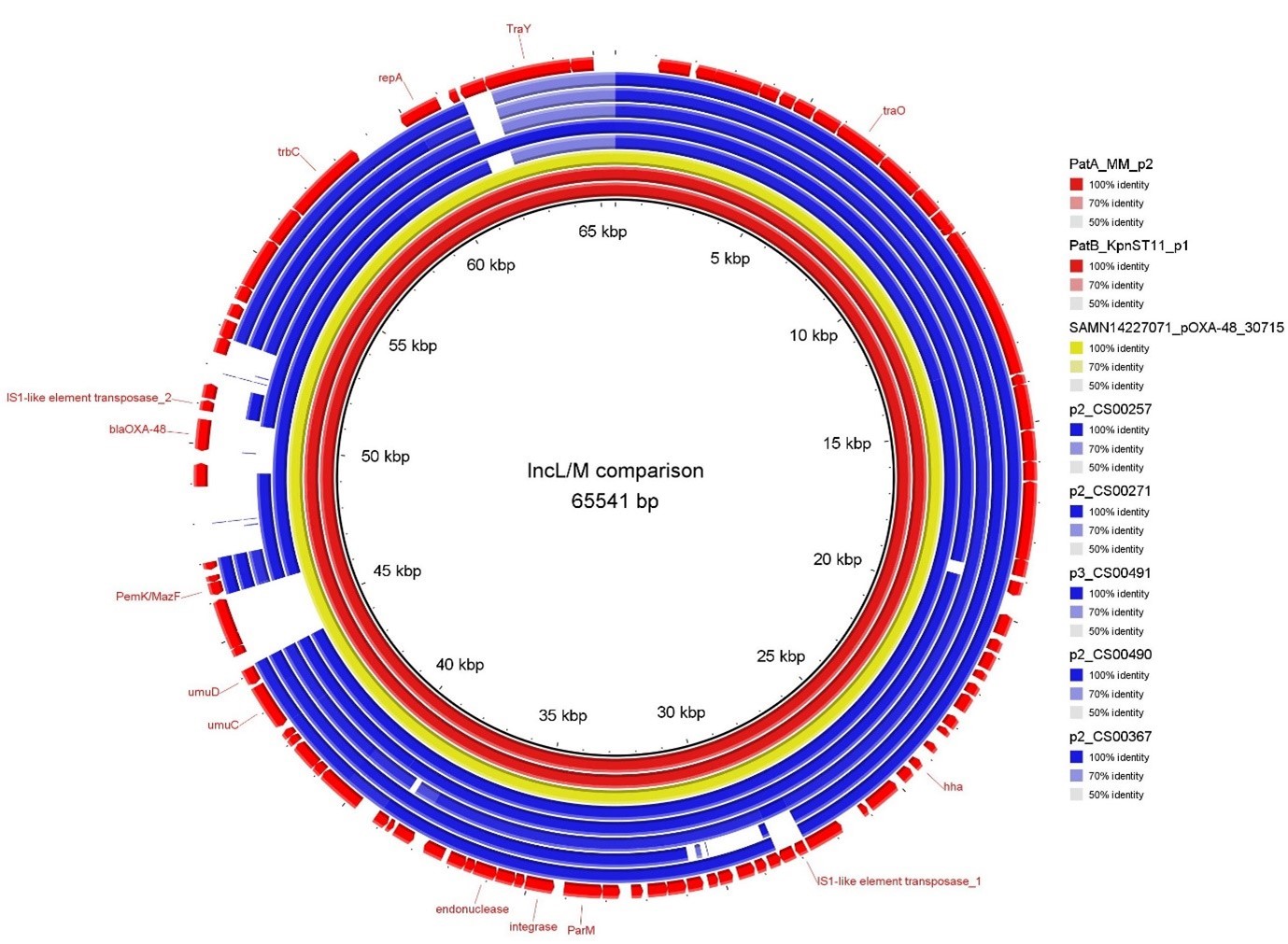

Supplement: Supplementary file 1 — Supplementary file1 (JPG 257 KB) Supplementary_Figure: Comparisons using BLAST analysis and BRIG software for imaging of IncL/M plamids. A) The IncL/M blaOXA-48 positive plasmids associated with patients from the Ukraine are colored in red, all other IncL/M plasmids found in our hospital in 2022 until April 2023 are colored in blue. As comparison, a reference plasmid from the NCBI database (GenBank accession number KX523901.1) was added (yellow) [file 15010_2023_2136_MOESM1_ESM.jpg]

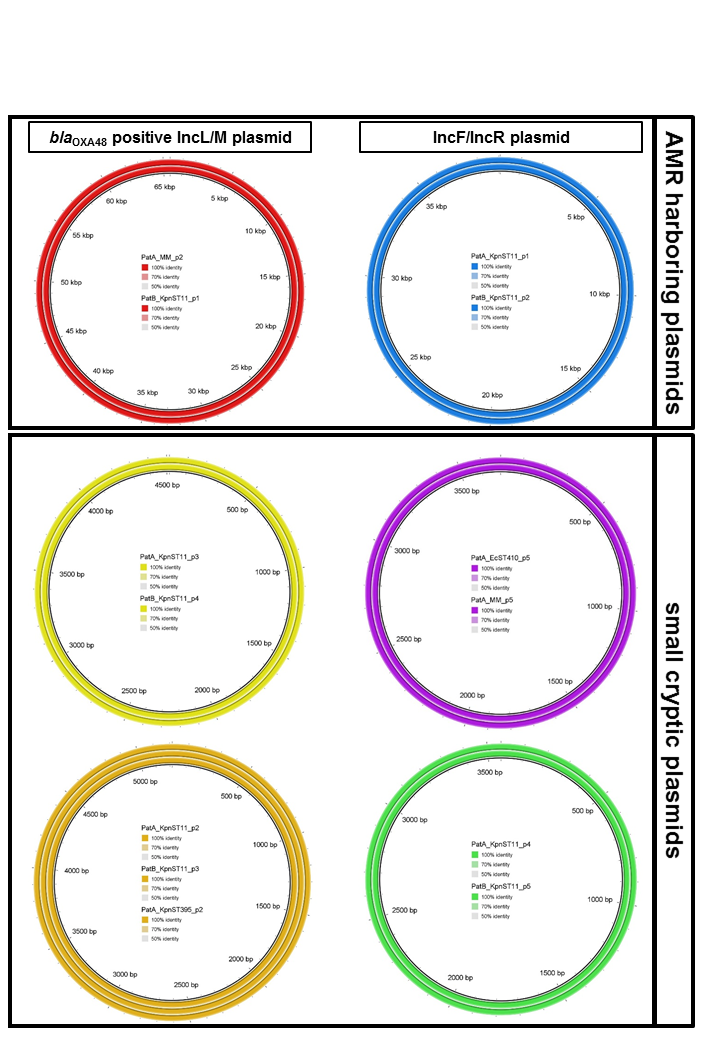

Supplement: Supplementary file 2 — Supplementary file2 (JPG 243 KB) Supplementary_Figure: Comparisons using BLAST analysis and BRIG software for imaging of IncL/M plamids. B) Blast comparisons of all detected plasmids. Color-coding was done according to Table 1: The IncL/M blaOXA-48 positive plasmids are colored red. An IncF/IncR plasmid is colored blue (PatA_KpnST11_p1 and PatB_KpnST11_p2). The four remaining plasmid groups colored in yellow, purple, orange and green belong to the SCP clusters (also see Figure 1) [file 15010_2023_2136_MOESM2_ESM.png]
